# Supplementary material for: Are carbon emissions trading and green financial instruments synergistic? -Comprehensive quantitative research based on content analysis
Source: PLoS One. 2024 Mar 7;19(3):e0298601. doi: 10.1371/journal.pone.0298601 (PMC10919601; doi:10.1371/journal.pone.0298601)
Supplement: S1 Data — (ZIP) [file pone.0298601.s001.zip › Data/地级市Z_F工作报告.zip!/╟α║ú/╬≈─■╩╨/└┤╫╘╣1⁄2╓┌║┼í╛┬φ┐╦╩2╛▌═°í┐_2014.docx]

各位代表：

　　现在，我代表市人民政府向大会作工作报告，请予审议，并请市政协各位委员和列席会议的同志提出意见。

一、2013年工作回顾

　　2013年，在省委、省政府和市委的坚强领导下，在市人大和市政协的监督支持下，市政府以党的十八大精神和习近平总书记系列重要讲话精神为引领，以科学发展观为指导，贯彻执政为民理念，坚持稳中求进、好中求快总基调，围绕建设人民满意的生活之城、幸福之城目标，全力稳增长、调结构、促改革、惠民生，全面完成了市十五届人大四次会议确定的各项目标任务。

　　————全力以赴稳增长、千方百计保就业，发展质量效益得到新提升。克服经济下行持续时间长，市场需求低迷，就业压力增大的复杂局面，大力调整经济结构，努力转变发展方式，统筹推进产业、企业、就业工作，国民经济运行稳中向好，就业工作取得较大成效。预计全年实现地区生产总值974.6亿元，全社会固定资产投资925亿元，地方公共财政预算收入67.1亿元，分别增长14%、32%和22.5%；城镇居民人均可支配收入和农民人均纯收入分别达到19750元和9010元，增长12%和15.5%；全年新增就业31486人，城镇登记失业率控制在3.04%。多项经济指标增速在全国26个省会城市中居于前列。

　　按照建设"国家循环经济发展先行区"的要求，加快发展特色优势产业，支持西宁经济技术开发区做大做强，促进县域工业发展，预计实现工业增加值435亿元，增长18%，其中开发区工业增加值占全市工业增加值比重达64.4%。围绕提升城市服务能力和增加就业，大力推进服务业综合改革试点，加快海湖总部经济、万达广场、多巴双寨综合物流园等服务业项目建设，实现社会消费品零售总额365亿元，增长15%。大力培育市场主体，实施民营经济倍增计划，新增规模以上工业企业46户；新培育各类市场主体20962户，增长24.3%，其中，中小微企业3160户、农民专业合作社467户、个体工商户17335个。推进创业型城市建设和"富民就业"工程，加大就业援助力度，加快各类创业孵化基地建设，全年安排大学生就业8026人，创业带动就业8103人,农村劳动力转移就业40万人次。

　　————攻坚克难治污染、凝心聚力抓生态，美丽西宁建设取得新成效。围绕 "国家生态文明发展先行区"建设，加大生态建设力度，节能环保资金投入达21.4亿元，增长176.7%。把大气污染综合治理作为改善生态环境、建设美丽西宁的突破口，实施攻坚行动，厉行治污先治吏，严格落实施工工地"五个百分百"要求，建立区县网格化环境监管模式，优化PM10和PM2.5运行监测，深入推进建筑扬尘、煤烟尘、机动车尾气、煤锅炉等29项专项治理，全年空气质量优良率达到60%以上，在全国74个大中城市排名位居中上游。创模通过省级预评估。

　　扎实推进"清水入城"工程，关闭了湟水河及其一级支流614个排污口和211处非法采洗砂场，加大污水处理厂及配套管网建设力度，完成了第一污水处理厂和城南污水处理厂提标改造工程，全省首座再生水厂即将投入运营，国控小峡口断面水质达标率83.3%。实施36个污染减排项目，污染物排放总量控制在省政府下达的指标之内。启动国家森林城市创建，投入2.96亿元完成林业生态工程32.9万亩，森林覆盖率达到30.6%；加快城市出口道路绿化美化，城区新增园林绿地70公顷，人均公共绿地达到10.6平方米，建成区绿化覆盖率达到38.5%。经过几代人几十年的不懈努力，大南山绿色屏障工程荣获住建部"中国人居环境范例奖"，成为西宁改善生态环境、建设美丽家园的典型示范！

　　————全神贯注保民生、统筹推进促和谐，人民生活质量有了新改善。倾听群众呼声，关心群众疾苦，把群众反映强烈的物价问题列为政府优先工作目标，贯彻落实省委"尖扎会议"要求，从生产、流通、市场监管、困难群众补助等方面多措并举，综合施策。新建冬暖式温棚2615栋、畜棚4000栋，建设市级露地蔬菜标准园1.1万亩，与四川彭州建立了稳定的蔬菜供销机制，在四川德阳建设蔬菜专供基地5000亩；抓好"农超对接"，建设平价商店50个，加大各类物资调运储备和市场投放力度，向市场投放平价粮油、蔬菜、肉等97500余吨；控制肉、菜等商品批零差率，减免摊位税费、发放补贴，努力降低流通成本；加大市场监管，严厉打击哄抬物价、囤积居奇等价格违法行为；为17.1万名生活困难群众累计发放物价补贴5023万元，最大限度减轻物价上涨对人民生活的影响。居民消费价格累计涨幅3.8%，实现了年初预定目标。

　　进一步加大"畅通交通"工程建设，新建过街天桥5座、停车场1个，5座跨河人行桥建成投运，更新超期服役公交车622辆，西宁被列入国家"公交都市"试点城市。通过路口渠化、设置公交专用车道、优化交通信号、退市还路等有效措施，主城区交通拥堵现象有所缓解。加强社会保障工作，提高保障水平，社会保障和就业投入达22.7亿元，城镇居民医保和新农合筹资标准均提高了70元；新开工建设城镇保障性住房35757套，17865户农村群众享受奖励性建房补贴和危房改造补助；"党政军企共建示范村"活动使8.6万群众受益，整村推进和易地扶贫地搬迁项目减少农村贫困人口5.1万人。努力调整财政支出结构，增加民生投入，财政用于民生支出达167亿元，占比80%以上，较上年多17.6亿元。年初向全市人民承诺的10大类58项办实事项目全面兑现！

　　————真情实意促公平、恪尽职守保稳定，社会建设取得新成效。坚持"科教兴市"战略，扎实推进国家创新型城市试点工作，6项科技成果获得青海省科学技术进步奖，我市连续6次荣获"全国科技进步先进市"称号。以办人民满意的教育为目标，积极推进"阳光招生"，完善"1+9+3"教育经费保障机制，制定加快普及十五年教育计划措施，教育预算支出达30.8亿元；投资16.4亿元实施教育布局调整、标准化学校、农村初中校舍、学前教育等项目，学有所教水平有了新提升。加快卫生事业发展，实施31所乡镇卫生院改扩建项目，基层医疗卫生服务能力进一步提高。推进文化体育旅游融合发展，共接待国内外游客1307万人次，增长15.9%，西宁机场旅客吞吐量突破300万人次；实现旅游总收入100.8亿元，增长34%。成功举办首届国际徒步节等活动，百姓大舞台文化传播公司荣获第五届全国服务农民服务基层文化建设先进集体称号。

　　推进"平安西宁"、"法治西宁"建设，全力做好维稳工作，依法打击各类违法犯罪活动。充分利用信息指挥中心和网格化平台，形成了责任明确、反应迅速、处置高效、监督有力的社会管理新格局。切实加强信访工作，有效化解社会矛盾。服务重心下移，新建34个社区综合服务中心、17个社区日间照料中心，699个老旧小区实现了物业管理全覆盖。加强食品药品监管，率先出台家庭式餐馆管理办法。更加重视大安全工作，加大食品、生产、交通、校园等领域的安全检查，切实做到安全生产管理"全覆盖"，安全隐患整改"零容忍"，大安监格局进一步巩固。

————突出重点攻拆迁、聚精会神搞建设，城市规划建设管理迈出新步伐。坚持规划引领，围绕推进重大项目建设、改善城市面貌、保障人民群众生命财产安全，针对征地拆迁、拆违打非这个难点、重点，敢于碰硬、善做工作、攻坚克难，完成了火车站站场及周边综合改造、中心广场北扩等多个国家和省市重点工程征地拆迁任务，促进了重点项目的顺利实施；异地安置拆迁北山危岩体辖区5万多名群众和苦水沟危险地段400余户居民，根除了该地区长期存在的环境脏乱差现象和地质灾害隐患；平稳推进沈家寨、杨家寨、北川河等片区征地拆迁，有效拓展了城市发展空间。全年共完成拆迁面积560.4万平方米，近两年累计拆迁1000多万平方米，超过前十年的总和。

　　大拆迁中民生在大改善、城市在大变化。全市各类房屋施工面积达1784万平方米，比"十一五"超出150余万平方米，一批功能完善、美丽大气的现代化小区相继建成，改善了群众居住环境；城镇基础设施建设得到长足发展，城市载体功能进一步提升，多巴新城启动建设，青海体育中心等建成使用，贵南路(桥)等一批城市道路建成通车，改造供排水管网92公里。实施各类建设项目654个，其中亿元以上新开项目59个；基础设施投资达143亿元，超过前2年的总和。把精细化理念贯穿于城市规划建设管理的全过程，《东部城市群西宁都市区战略规划》、《西宁新区发展规划》等一批重点规划相继完善和实施；推进精细管理与流程再造，强化市容环境整治，"二次创卫"顺利通过国家复审，保持了国家卫生城市的荣誉称号！

　　————坚定不移抓改革、毫不动摇扩开放，体制机制建设有了新进展。始终把改革作为动力之源，认真开展第七批行政审批项目清理工作，取消和调整行政审批项目26项，行政审批提速提效。加快城市基层管理体制改革，开展社区服务体制改革试点，精简管理层级，优化整合资源，社区在城市管理和居民服务中的基础性作用得到充分发挥。以政府购买服务形式，在全省率先开展了居家养老服务试点。深化国库集中支付制度改革，直接支付比例达95%以上。推进农村户籍制度改革，转户农民达29.3万人，全市城镇化率达68.2%。深化县级公立医院综合改革，巩固完善"先住院后结算"医疗服务模式，基本药物覆盖率、零差率均保持在100％。市直国有文艺院团改革平稳有序，组建了西宁艺术剧院有限公司，文化市场竞争力明显提升。充分利用"青洽会"、"城洽会"等会展活动，进一步拓展对外交流合作，实施招商引资项目477个，到位资金562亿元。成功引入中信银行等机构，全年重点项目融资额达135亿元，西宁地区各项贷款余额2698.3亿元，占全省贷款余额的80.3%，金融服务实体经济的作用更加明显。

　　一年来，我们全力攻坚全国文明城市创建，顺利通过中央文明办年度城市文明程度指数测评，排名和位次有了新提升，市民素质和城市文明程度明显提高，再次获得省级文明城市称号。确定了"两年强基础、四年见成效、六年创先进"的民族团结进步先进区创建目标，各方面力量得到有效凝聚。双拥共建和国防后备力量建设进一步加强，驻军（警）部队对西宁城市建设和各类创建活动作出了积极贡献。同时，统计、工商、气象、供销、民族宗教、外事侨务等工作得到加强，妇女、青少年、残疾人、红十字会等事业取得长足发展。值得一提的是，在伟大的玉树灾后重建中，我市竭尽全力，累计完成投资11.7亿元，58个援建项目全面完成，获得"城乡居民住房建设工程质量进度奖"等9项奖励，谱写了新杂多建设的光辉篇章，浇筑了西宁人民和玉树人民的深厚友谊！

　　我们坚持建设服务政府、法治政府，健全完善科学民主决策制度，努力转变政府职能，贯彻落实中央和省委、省政府、市委有关规定，整治"慵懒散奢"，工作作风进一步好转。自觉接受人大法律监督和政协民主监督，自觉接受人民监督，定期向人大及其常委会报告工作，及时向政协通报情况，密切与各民主党派、工商联、人民团体保持联系，广泛听取社会各界人士意见建议。及时办理人大代表议案建议和政协提案，办复率均为100%。全面落实党风廉政建设责任制，深化廉政文化"六加四"西宁模式和"一岗双责"报告制度，改革政风行风评议办法，在全省率先开展"向人民汇报、请人民评议"现场直播活动，建立农村"三资"网络监管平台，健全村务监督委员会工作机制。强化行政监察和审计监督，加大行政问责力度。公开"三公"经费，严格控制机关公用经费支出，行政运行成本明显降低，效率进一步提高。

　　各位代表，回首过去的一年，我们深切体会到，坚持党的领导是做好政府工作的根本保证，坚持人民至上是做好政府工作的重要理念，坚持改革创新是做好政府工作的不竭动力，坚持敢于担当是做好政府工作的重要要求，坚持实干兴市是做好政府工作的最大追求，坚持清正廉洁是做好政府工作的重要前提。这也是我们必须长期坚持、一以贯之的行政理念。发展令人振奋，成绩来之不易，饱含着省委、省政府的深切关怀，凝聚着全市各族人民的共同努力。在此，我代表市政府向各位人大代表、政协委员，向各民主党派、工商联、无党派人士和各人民团体，向奋斗在全市各条战线、各行各业的建设者和劳动者，向中央驻宁单位、省直各部门、人民解放军驻宁部队指战员、武警官兵和公安干警，向所有关心、支持西宁建设和发展的各界人士，致以崇高的敬意和衷心的感谢！

　　同时，我们也清醒地看到，我市经济社会发展中不平衡、不协调的问题和矛盾依然突出：城市基础设施还不完善，加快提升承载能力任务艰巨；市场体系发育较慢，市场主体量少质弱，深化改革任务艰巨；县域经济实力较弱，城镇化层次较低，加快农村基本公共服务建设任务艰巨；经济结构重化工特征明显，保持经济快速增长与完成节能减排任务矛盾突出，建设生态文明新型城市任务艰巨；受国内市场价格和我市"从全国买，向全省卖"特殊因素影响，保持物价总水平相对平稳任务艰巨；个别部门的责任意识还不强，一些干部的工作作风还不够扎实，转变工作作风任务艰巨。对此，我们将高度重视，采取切实有效的措施逐步加以解决。

二、2014年主要目标和工作任务

　　2014年，是贯彻落实党的十八届三中全会精神、全面深化改革的第一年，也是顺利完成"十二五"规划、推进"两城"建设的关键一年。做好今年的工作，任务艰巨，意义重大。

　　根据市委十三届八次全体会议精神，政府工作的总体要求是：以党的十八大、十八届三中全会精神和习近平总书记系列重要讲话精神为指导，深入开展党的群众路线教育实践活动，全面落实省委十二届五次全会精神，谋发展之要，行惠民之举，务固本之实，强执政之基，在改革上当先锋，在发展上争先进，在建设上显特色，在民生上有作为，在创建上出亮点，在和谐上作表率，在更高层次、更大范围为人民群众谋更多福祉。

　　经济社会发展的主要预期目标是：实现"三跨"、"两增"、"两控制"、"一确保"。即：地区生产总值和固定资产投资跨千亿，农民人均纯收入跨万元，分别增长12.5%、25%和15%；地方公共财政预算收入增长15%、城镇居民人均可支配收入增长10%；居民消费价格涨幅和节能减排控制在省定目标以内；确保空气质量优良率提高到65%以上。

我们在确定今年的指标时，适度下调了生产总值等预期增幅，继续保持物价调控、城乡居民收入等民生指标相对稳定。体现了中央和省委关于提高发展质量和效益的要求，符合我市实际需要，有利于把各方面的注意力引导到调整结构、提质增效、改善生态、惠及民生上来。

实现上述目标，做好今年的政府工作，关键是贯彻"坚持正确方向、全面深化改革、奋力打造"三区"、建设全面小康"战略，把握稳中求进、改革创新、提质增效，推动全面深化改革，以改革促创新、促发展；把握全面建成小康社会的目标要求，努力保持经济平稳较快增长；把握发展由速度向质量效益转变的必然趋势，着力打造西宁经济"升级版"；把握生态在全局工作中的战略地位，着力打造美丽西宁"升级版"；把握发展成果惠及人民的根本目标，着力打造民生改善"升级版"；把握市场和政府的关系，积极营造健康稳定的发展环境。重点抓好以下七个方面的工作：

　　第一，更加注重激发社会活力，率先深化各项改革。以更大勇气和智慧，准确、有序、协调推进改革，在重点领域和关键环节取得突破，形成符合西宁实际的制度体系，在全面深化改革上走在全省前列。

加快经济体制改革。积极培育市场主体，继续实施民营经济倍增计划，进一步改善发展环境，全面清除各种市场壁垒，建立有利于市场主体加快发展的制度，鼓励、支持和引导民营经济发展，使民营经济总量占全市经济总量比重持续提升。深化国家服务业综合改革试点，出台服务业发展指导目录，细化政策支持措施。抓好国资部门经济体制改革，抓紧研究公交公司、城投公司、湟投公司等企业深化改革具体措施，确保取得实效。积极发展国有资本、非公有制资本等相互融合的混合所有制经济，推进国有企业股权多元化，建成一批混合所有制企业。完善预算管理制度，试编财政三年滚动规划，继续推进"营改增"试点。深化行政体制改革。按照中央和省上统一部署，完成政府职能转变和机构改革任务。继续深化行政审批制度改革，再精简一批行政审批项目。进一步完善行政服务中心功能，推行网上审批试点，建立公开、高效、快捷的工作机制，为群众办事提供更多便利。深化城市社区服务体制改革试点，完善社区公共服务中心职能和运行机制，满足群众多样化、多层次、多功能的服务需求。加快制定政府向社会组织购买公共服务的具体意见和办法，创新完善公共服务供给模式，在环卫、绿化、教育、就业培训等领域采取公开招标方式向社会购买公共服务。推进城市建设管理体制改革。出台加强城市规划建设管理工作意见，建立产业、园区规划与城市总体规划的衔接协调机制，推进产城融合发展。实行城市管理相对集中执法，着力构建"大城管"体制。创新城市建设融资机制，引导民间资本通过产业基金、股权置换、特许经营等多种途径进入基础设施、基础产业和公共服务领域，使社会资本成为推动城市建设与发展的重要支撑。实施城乡一体化改革。制定深化土地承包经营权流转改革方案，鼓励发展新型农业经营主体。完善农村宅基地管理制度，修订并实施村民住房建设管理办法。加快生态文明改革。进一步完善环境污染区域联防联治和重污染天气应对机制，实行更加严格的环境保护监管制度。围绕创建国家森林城市，加快林业产业市场化改革。推进民生事业改革。深化户籍制度改革，有序放开户籍限制，加快推进农业转移人口市民化,深化教育、科技、文化、卫生、食品药品监管等体制改革。

　　第二，更加注重质量效益，努力推进产业转型升级。坚持把调结构、稳增长作为主攻方向，把推动经济转型升级放在突出位置，发挥好财税杠杆调控作用，强化金融支撑能力，增强创新驱动能力，固本强基发展实体经济，有效增强经济发展后劲，在创建 "循环经济先行区"上走在全省前列。

　　推进工业转型升级。着力优化工业结构、培育优势产业，确保工业投资完成460亿元以上，工业增加值增长16%。大力支持开发区建设，协调解决招商引资、项目建设等环节的困难和问题，实施好两个千亿元、两个五百亿元产业基地建设，抓好227个重点支撑项目建设和110个重点招商引资项目培育工程，建设50个创新平台，积极推进光伏制造、铁合金、中藏药兼并重组，提升产业发展水平。加快实施煤基多联产、高端铝合金型材、碳材料等一批产业链延伸项目，推进高倍聚光光伏发电系统、锂电正极材料等项目，形成新的产业规模。优化融资和服务环境，抓好大通北川500亿元产业基地建设。加强中小微企业扶持，形成一批科技型、成长型企业,年内新培育中小微企业3500户以上。实施好"123科技支撑项目"和33个省级重大工业技术进步项目，淘汰一批落后产能，推进企业技术创新，加速产业转型升级。

　　提升第三产业水平。以国家服务业试点城市建设为抓手，推进服务业集聚区建设和文化体育旅游融合发展，促进服务业提质增效, 力争第三产业增加值增长11%。突出以高端商贸设施和城市综合体建设为重点的服务业集聚区建设，抓好国家共同配送试点城市申报工作，推动西宁机场增开国内外客货运航线。推进海湖新区核心区、水井巷中央商务区、环新宁广场高端商务区、建国综合商务区、双寨物流园区等功能区建设，启动香格里拉商业综合体、上海绿地集团等项目建设。围绕改造提升传统服务业和培育新型服务业，打造一批特色街区和支撑性项目。全面整合文化旅游体育资源，推进藏医药博物馆群等5A级景区创建和西宁游客集散中心建设，启动塔尔寺大景区建设，建好北山烟雨景区。大力发展电子商务，引进大型电商龙头企业，鼓励中小企业参与投资，搭建网上商城，积极拓展市场。全面推进"智慧城市"建设，促进信息消费。加快会展经济发展步伐，办好"城洽会"、夏都国际论坛暨世界清凉城市大会等品牌节会，加强项目推介，力争招商引资到位资金700亿元以上。

　　做强高原现代农业。确保粮食安全，稳定粮油生产，以提高菜篮子生产保障能力为重点，推进高原现代都市农业发展。着力构建新型农业经营体系，集中连片发展设施种养业，积极扶持和培育家庭农牧场、种养大户、农民合作组织，引导农民加大土地流转力度。严格落实菜田"红线"保护、露天蔬菜补贴等办法，增强菜篮子主要产品的供给保障能力，新建冬暖式温室2500栋，改造提升旧温室1000栋，力争蔬菜产量达到82万吨；种植优质饲草45万亩，新建畜棚4000栋、各类养殖基地45个，肉、蛋、奶产量达到7.82万吨、1万吨、14.5万吨。

　　第三，更加注重城乡统筹，不断增强载体服务功能。把握国家推进城镇化的政策机遇，坚持"扩市提位"战略，加快旧城改造，推进新区开发，完善基础设施，进一步增强西宁作为青藏高原门户枢纽和中心城市的支撑力和影响力。

　　做深做细做优规划。充分把握战略观、人本观、山水观、文脉观、法治观，强化规划龙头作用。围绕合理布局、节约土地、集约发展的原则，使城市规划建设有追求、有品位、有规矩、有特色。加快重点片区、重要地段及成片开发区域、重要街区、重要公共服务设施项目的修建性详细规划编制。全面推行城市规划三维审批和城区网格化管理，研究制定地下管线综合管理办法，出台加强规划建设管理工作的指导意见。

　　开辟更大发展空间。加快西宁新区规划审批，推进昆仑大道、柴达木路西延段等重大基础设施建设，拉开新区骨架。完善提升建国路、中心广场、新宁广场、海湖、北山"五大片区"建设。与火车站站改工程同步推进建国路片区建设；按照规划方案，精心实施中心广场片区建设，打造城市新亮点；加快新宁广场片区提升改造步伐，形成现代商贸休闲新区域；完善海湖新区综合服务功能，集聚人气、初具规模；机场高速北侧以北山片区建设为依托，形成永久性绿色生态景观长廊，南侧重点打造新型物流商贸区。加快南川、西川、北川"三川"建设步伐。南川片区抓好拆迁后的土地整理和道路供排水、电网等基础设施建设，尽快形成开发条件；西川片区重点在新城基础设施、重点开发项目招商方面取得实质性突破；北川片区做好生态水城文章，完成水系治理，搞好绿化、引进大商。同时，全面完成沈家寨、门源路、曹家寨等重点区域的征地拆迁任务，为开发建设开辟新的空间。

　　加强基础设施建设。紧紧抓住国家实施"丝绸之路"经济带、向西开放等发展机遇，推进东部城市群建设，增强城市载体功能，提高城市现代化水平。加快城市电网、供热和供排水管网改造，努力实现天然气城区全覆盖。促进引大济湟工程顺利实施。配合做好火车站站改、兰新铁路第二双线、南绕城公路、西塔高速改线等项目工作。实施好110个城市基础设施建设项目，力争完成投资220亿元。

　　加快城镇化建设步伐。坚持走新型城镇化道路，提高城镇建设水平，全力打造有历史记忆、地域特色、民族特点的美丽城镇。完善三县重点城镇发展建设规划，坚持以市定标，实施"大县城"发展战略，加快多巴城区建设步伐，支持三县县城和城关、长宁、新庄、李家山、大华等基础较好、人口相对集中、发展潜力较大的乡镇加快建设，整治县城、小镇及主要交通沿线环境秩序，建设美丽县城、美丽小镇、美丽村庄。注重用产业发展支撑城镇建设，统筹规划建设县域产业园区、城镇新区、农村新型社区和农民新村，依托当地资源优势，大力发展绿色产业，有序推进工业空间布局集中。围绕产业链延伸和园区基础设施抓好招商引资工作，力争形成一批带动性强的产业项目，促进产业与城镇化发展深度融合。适应城镇化发展需要，推动行政区划调整。加快推进公共服务均等化，不断完善农村教育、医疗、文化、体育等公共服务设施，健全基本医疗、养老等保障机制，加快农村服务大厅和村级信息服务站建设，使广大农民群众享受更多的公共服务。

　　第四，更加注重以人为本，努力改善民生福祉。坚持不懈解决城乡群众最关心、最迫切的民生问题，实施以区县为单位全面建成小康社会行动方案，让人民群众的生活更加美好。

　　千方百计为群众提供更加充分的就业机会。坚持就业优先战略，不断培育企业、延伸产业，开发更多就业渠道和就业岗位，确保劳动力充分就业。启动运行市级创业孵化园，建成四区大学生创业孵化园，加快三县创业实训基地建设。进一步加强技能培训，为民众创业就业提供便利。落实好促进创业、就业的各项优惠政策，鼓励高校毕业生、退伍军人、失地农民自主创业和灵活就业，新增就业3万人以上，让更多的人群通过辛勤工作，创造美好生活！

千方百计为群众提供更高水平的社会保障。把人人享有基本社会保障作为根本目标，继续提高城乡基本养老保险覆盖率和企业退休人员基本养老金、城乡低保及优抚对象补助标准。执行好城乡居民基本医保政策和城镇职工、城乡居民基本医疗保险分级诊疗等制度，完善城乡困难群众大病医疗救助政策，切实减轻群众就医负担。关注弱势群体生活，发展残疾人事业，做好公益慈善工作。积极应对人口老龄化挑战，大力发展养老服务业，加强城市社区养老机构和农村养老设施建设，培育发展医疗护理、康复理疗、保健养生等新型服务，让老年人享受安康幸福的生活。

千方百计为群众提供更加完善的住房体系。促进保障性住房建设和房地产市场健康发展，建立"保障+市场"的双轨住房供应机制，加强基础设施配套，完成棚户区改造39971套，力争2015年基本消除棚户区;完成城镇保障性住房建设47366套、农村危房改造6000户、农村奖励性住房10000户，从根本上改善困难群众居住条件。准确把握房地产市场宏观调控导向，引导市场合理投资，促进房地产市场稳定健康发展，力争完成投资240亿元以上。

千方百计为群众创造更加顺畅的交通出行。进一步完善城市路网布局规划，全面推进世行交通项目五四西路西延段、西城大街工程建设，完善城市路网结构。持续推进"畅通交通"工程，认真开展国家"公交都市"试点，推进快速公路、快速公交建设，再建成一批人行桥、过街天桥、停车场，努力缓解城市拥堵现象。年内启动建设轻轨1号线试验段，积极推进智能交通信息系统建设，争取进入全国"一卡通"互联互通试点城市，提高城市公共交通营运能力和服务水平，为市民提供更加顺畅的交通出行。

千方百计为群众提供更加充足的市场供应。将保供稳价作为民生政府的关键担当，在源头上抓增量，在流通中减环节，在市场中抓管理，继续采取政府补助、批零差率控制、物价专项整治等措施，切实抓好四川德阳蔬菜专供基地建设，落实好四川彭州和海东市蔬菜购销协议，充分发挥青藏高原农副产品集散中心的辐射调控作用。加快各城区次中心或小型市场建设，再建一批平价商店，继续推进早市进社区和"农超对接"，努力保障市场供应充足、物价水平基本稳定，确保居民消费价格指数涨幅控制在大多数群众承受范围。

千方百计为群众创造更加有效的扶贫机制。坚持专项扶贫、行业扶贫、社会扶贫、援青扶贫的"四位一体"大扶贫格局，创新扶贫开发体制机制，着力增强贫困地区"造血"功能。充分发挥县级政府在扶贫攻坚中的主体作用，整合涉农资金，促进扶贫政策、规划、资金和帮扶"四到村、四到户"。抓好85个贫困村连片开发整村推进，完成43个贫困村易地搬迁，减少贫困人口5万人。

　　第五，更加注重社会发展，不断满足群众公共服务需求。坚持把社会事业发展摆到更加重要的位置，努力办好人民群众最直接、最关心的事，把群众利益实现好、维护好。

　　努力提供优质教育资源。全面实行"阳光招生"，实施普及十五年教育行动计划措施，加快普及学前三年和高中阶段教育，努力推进教育均衡发展。投资17.7亿元实施85项教育建设项目，抓好西宁城市职业学院组建、农村学前教育、城区示范性幼儿园、中小学生社会实践基地等项目建设，启动新一轮城区中小学校布局调整项目，完成十四中、特校迁建等项目。加强教师队伍建设，提高师德水平和业务能力，增强教书育人的荣誉感和责任感。

　　大力促进卫生事业发展。积极推进"健康西宁"建设，大力发展公共卫生事业，进一步完善国家基本药物制度和基层医疗卫生机构运行机制，加强疾病预防控制，夯实人民群众的健康幸福基础。加强市级重点学科和县级医疗机构能力项目建设，抓好社区卫生服务机构规范化建设，开展城乡基层医疗卫生一体化管理和居民家庭医生签约服务工作。加快海湖医院、市第一人民医院综合楼、市第三人民医院儿童诊疗中心等项目建设，不断改善就医条件。深入开展"和谐医患关系示范区"创建活动，妥善处理和化解医患矛盾，让群众享受到舒心的就医和温馨的服务。

　　不断提升公共文化服务水平。以打造文化品牌为重点，以传统节会和重大赛事活动为平台，实施市、县两级文化品牌战略，大力发展文化事业和文化产业。继续提升以百姓大舞台、社区文化艺术节等为载体的群众文化品牌内涵，不断扩大西宁青年电影展、国际舞蹈节等重大文化活动的知名度和影响力。进一步拓展对外文化交流渠道，积极争取承办更多有影响的全国性、国际性文化活动。开展国家级公共文化服务体系示范区创建，实施四级公共文化服务体系和基层文化服务场所规范化、标准化建设，让更多群众享受到丰富的文化生活。

　　第六，更加注重生态文明，全力建设美丽西宁。围绕建设"国家生态文明先行区"战略，下大气力做好治气、治水、治山和减排文章，着力推进绿色发展、循环发展、低碳发展，筑牢生态基础，让城市融入自然。

　　深入治气。坚持不懈抓好大气污染治理，全面完成天然气管网覆盖范围内809蒸吨燃煤锅炉改造，基本完成餐饮服务单位煤油烟污染治理。强化对建筑和拆迁工地、渣土车辆等扬尘污染和机动车尾气污染治理，完成油气回收改造和"黄标车"淘汰任务。加大环境执法力度，严厉打击偷排漏排污染物等违法行为，以更加坚定的态度、更加有力的措施和更加扎实的工作，使空气质量优良率明显提升，让人民群众呼吸上新鲜的空气，看到湛蓝的天空！

　　持续治水。继续推进"清水入城"工程，加强污水处理厂提标改造、再生水利用、垃圾填埋场管理、饮用水水源地保护等工作，加快第四、第五污水处理厂及配套管网工程建设，启动第六污水处理厂项目，抓好城市排水箱涵建设，实施雨污分流工程，提高城区污水收集率，确保国控小峡口断面水质稳定达标。推进西宁环境综合治理利用世行贷款项目评估及签约工作，争取早日实施。严格水资源管理，推进全国水生态文明城市建设试点工作，加强黑泉水库周边环境综合整治及水源涵养保护。全面实施北川河综合治理，完成南川河防洪及河岸景观绿化等工程，建立西宁湟水国家湿地公园，力争把三河六岸建设成集生态防护、休闲旅游、文化展示、自然恢复功能为一体的绿色景观生态廊道。

　　不懈治山。围绕创建全国森林城市目标，坚持不懈推进大南山生态绿色屏障、城市绿地、主题公园和休闲绿地等林业工程建设，继续实施"四边"绿化、宁湖景观改造等重点工程，完成造林34.5万亩。加大主城区街头小绿地、小公园建设力度，打造西宁环城森林公园，新增园林绿地120公顷，让城市树多花多、绿意盎然、花香满城、充满生机。

　　节能减排。大力发展循环经济，推进结构减排、工程减排和管理减排，全面巩固和提高创模已达标的各项指标，强力整改尚未达标的目标。结合产业结构优化升级，鼓励企业技术改造，加强节能减排重点工程建设，推进绿色建筑创建和低碳交通运输体系建设。以工业园区为主战场，实施节能环保产业园建设。坚决实行空间、总量、项目"三位一体"环境准入制度，严格控制新上高耗能、高污染和资源性项目，确保完成年度减排任务。

　　第七，更加注重发展环境，深入推进城市创建。坚持以创建为抓手，充分发挥广大群众的主体作用，聚焦问题、聚焦作风、聚焦民生，以创建促经济持续发展、社会文明和谐。

　　加强民族团结进步。实施民族经济发展项目，加大少数民族群众技能培训和就业创业，推进社会保障体系和农村综合服务设施建设，提升民族地区公共服务水平，切实增加群众收入。继续加大先进单位、示范点创建力度，打造一批富有特色的示范品牌，以点带面，集点成面。依法加强宗教事务管理，全面贯彻落实民族政策，促进民族团结和宗教和睦，在"民族团结进步先进区"创建中发挥引领和表率作用。

　　做好创城迎检工作。针对创城工作中的薄弱环节和存在的问题，以更加坚定的信心、更加主动的姿态、更加务实的作风、更加精细的准备，落实好公共基础设施建设管护、环境综合整治、文明交通建设、公共秩序优化、社会文化环境净化、公民素质提升等工作，培育和践行社会主义核心价值观，力争创城工作在"冲刺年"再上新台阶，为"文明青海"建设做出应有的贡献。强力攻坚国家环保模范城市创建，争取顺利通过环保部技术评估，使西宁成为全省环境保护的表率。坚持"建卫日"制度，持续巩固创卫成果，营造卫生、整洁、优美的城乡环境。

深入推进平安创建。推进"法治西宁"、"平安西宁"建设，认真学习发展"枫桥经验"，不断夯实基层基础工作，从源头上预防和解决社会矛盾问题。建设安全城市，加强社会治安综合治理，逐步完善高清视频监控系统，严厉打击各类违法犯罪活动，不断增强群众的安全感。畅通群众利益诉求表达渠道，全力化解信访积案。实施《法治西宁建设纲要》，依靠法治力量和法治方式调处社会矛盾，努力营造法治社会人人参与、全民共建的良好氛围。加强全方位安全检查和监督管理，加大隐患排查整治，完善防范措施，建立长效机制，持续巩固大安监格局。创建国家食品安全示范城市，扎实推进食品药品安全监管城乡一体化建设，依法严厉打击各种食品药品安全违法行为，用最严谨的标准、最严格的管理、最严厉的处罚、最严肃地问责，让制假售劣者和违法违规者无处藏身，确保人民群众吃上更加安全的食品，用上更加放心的药品！

三、加强政府自身建设

　　各位代表，改革发展稳定的重任和人民的期盼，对政府自身建设提出了更新、更高、更严的要求。我们必须不断顺应人民群众的愿望和要求，从严治政、从严治吏，对事业态度严肃，对干部要求严格，对工作纪律严明，对违规坚决严惩，在深化改革上不畏难、在解决问题上不停步、在改善民生上不懈怠、在做好工作上不慵懒，不断解放思想、开拓进取，弘扬奋勇争先、干事创业的优良政风，积极转变政府职能，全面履行政府职责，努力建设为民、务实、高效、廉洁的政府。

把为民的宗旨体现好。坚持以人为本、执政为民，进一步强化宗旨意识、问题意识、机遇意识、责任意识，深入开展群众路线教育实践活动，不断转变职能、改进作风、优化服务，努力提升政府工作效率和水平。强化服务企业、服务基层、服务群众意识，深入企业、融入农村，努力为基层排忧解难，多办顺民意、解民忧、暖民心的实事。继续开展"向人民汇报、请人民评议"活动，倾听人民呼声，接受人民监督。

　　把务实的作风发扬好。强化敢于作为、善于作为、有所作为的意识，把"争先进位、争创一流"贯穿工作始终。围绕破解难题创新思路，围绕优化服务创新办法，围绕激发活力创新机制。进一步转变作风，大力推行"一线工作法"、"现场工作法"，加强行政监察，严格落实限时督办、效能监察和绩效评价制度，以严肃地问责问效推动工作落实。

　　把依法的要求落实好。坚持依法决策、科学决策、民主决策，规范行政执法行为，提高行政执法水平和效能，做到照章办事，恪守原则，心怀敬畏，慎重用权，努力创造良好发展环境、提供优质公共服务、维护社会公平正义。认真接受人大法律监督和政协民主监督。认真执行市人大及其常委会的决议和决定，切实做好代表意见、建议的办理。落实重大行政决策程序规定，扩大公众对政府决策的有效参与。

　　把清廉的律条遵守好。坚持用制度管权、管事、管人，促使干部敬畏权力、慎用权力。继续加大对招投标、工程建设、政府采购等重点领域的资金监管和政府性投资项目的监察审计，严肃查处各类违法违纪案件，严肃查处工作中执行不力、落实不力的人和事。严格落实中央和省、市委关于改进作风的一系列制度措施，厉行勤俭节约，反对铺张浪费，严格控制"三公"经费，降低行政成本，把有限的资金更多地用在发展经济、改善民生上，树立勤政、清廉的良好形象。

各位代表，加快推进科学发展，全面建成小康社会，是时代的呼唤、人民的重托、历史的责任。让我们更加紧密地团结在以习近平同志为总书记的党中央周围，在省委、省政府和市委的坚强领导下，携手全市各族人民，攻坚克难，勤勉工作，为加快建设人民满意的生活之城、幸福之城而努力奋斗！
